# Comprehensive analysis of heterojunction compatibility of various perovskite solar cells with promising charge transport materials

**DOI:** 10.1038/s41598-023-46482-5

**Published:** 2023-11-03

**Authors:** Shayan Tariq Jan, Muhammad Noman

**Affiliations:** 1 U.S.-Pakistan Center for Advanced Studies in Energy, University of Engineering and Technology, Peshawar, Pakistan; 2 Department of Energy Engineering Technology, University of Technology, Nowshera, Pakistan

**Keywords:** Solar cells, Solar cells, Software

## Abstract

The allure of perovskite solar cells (PSCs), which has captivated the interest of researchers, lies in their versatility to incorporate a wide range of materials within the cell’s structure. The compatibility of these materials plays a vital role in the performance enhancement of the PSC. In this study, multiple perovskite materials including FAPbI_3_, MAGeI_3_ and MASnI_3_ are numerically modelled along with the recently emerged kesterite (CBTS, CMTS, and CZTS) and zinc-based (ZnO and CdZnS) charge transport materials. To fully explore the potential of PSCs and comprehend the interplay among these materials, a total of 18 PSC structures are modeled from different material combinations. The impact of band gap, electron affinity, absorption, band alignment, band offset, electric field, recombination rate, thickness, defects, and work function were analyzed in detail through a systematic approach. The reasons for varying performance of different PSCs are also identified. Based on the simulated results, the most suitable charge transport materials are CdZnS/CMTS for FAPbI_3_ producing a power conversion efficiency (PCE) of 22.05%, ZnO/CZTS for MAGeI_3_ with PCE of 17.28% and ZnO/CBTS for MASnI_3_ with a PCE of 24.17%.

## Introduction

Photovoltaic (PV) cells are semiconductor devices that harness the photons from the sun converting them into electricity. These cells play a pivotal role in sustainable energy solutions, offering a clean and renewable alternative to fossil fuels. The PV cells have seen significant evolution, leading to categorizations into distinct generations. The first generation primarily includes crystalline silicon cells, with monocrystalline silicon (Mono-Si) cells known for their high efficiency and unique rounded appearance, and polycrystalline silicon (Poly-Si) cells, which are more affordable but slightly less efficient, recognizable by their blueish hue^1–3^. The second generation introduces thin-film solar cells, such as the amorphous silicon (a-Si) cells used in small electronic devices, cadmium telluride (CdTe) cells with their cost-effective production, and copper indium gallium selenide (CIGS) cells that promise higher efficiency. The third generation brings advanced technologies like the flexible organic photovoltaic (OPV) cells, rapidly advancing perovskite solar cells (PSCs)^4, 5^, dye-sensitized solar cells (DSSC) that employ dyes for sunlight absorption, and quantum dot solar cells that can be tuned for specific solar spectrum absorption^6–8^. Additionally, tandem or multi-junction cells stack multiple solar cell materials to maximize efficiency by targeting various parts of the solar spectrum^9^. While the widely adopted first-generation cells dominate large-scale solar installations, the subsequent generations provide versatility, potential cost savings, and innovative applications, with the choice of solar cell often dictated by application needs, budget, and efficiency goals.

In recent years, the new hot technology that has emerged from the PV industry is the PSC. The materials that have the crystal structure of ABX_3_ are classified as perovskites^10^. The “A” represents the cations of organic, inorganic, or hybrid molecules^11^. The “B” represents the metal cations of period VI metals^12^. While the “X” represents halide anions^13^. The PSCs have attracted the attention of the research community due to their tunable band gap, high optical absorption, excellent carrier mobility, and suitable electron affinity. The most prominent PSCs are the methyl ammonium lead iodide (CH_3_NH_3_PbI_3_) which has achieved a remarkable power conversion efficiency (PCE) of 25.7%^14^.

The CH_3_NH_3_PbI_3_-PSC suffers from thermal instability due to the material’s low thermal conductivity^15^. The methyl ammonium (MA) in the perovskite is organic in nature^16^. The low conductivity causes heat to build up in the bulk of the material, which in turn degrades the MA rapidly, leading to the collapse of the perovskite crystal structure^17^. This causes the leakage of lead (Pb) into the surrounding environment which is toxic in nature.

The significant advantage of perovskite materials is that the elements can be replaced with alternatives in the ABX_3_ crystal while still maintaining its PV properties. The “A” cation of the perovskite material can be replaced with formamidinium (FA—HC(NH_2_)_2_)^18^. The FAPbI_3_ has higher thermal conductivity than the MAPBI_3_ and has shown superior stability than its counterpart while achieving a PCE of more than 20%. Similarly, the “B” cation can be replaced with tin (Sn) or germanium (Ge)^19^. Both materials are environmentally friendly and have shown to boost the thermal conductivity and stability of the cell when deployed in the perovskite material (MASnI_3_ and MAGeI_3_)^20^. The Sn-PSC has achieved a PCE of more than 14%^21^. On the other hand, little progress has been made in Ge-PSC due to difficulty in its fabrication. However, theoretical analysis through density function theory and numerical modeling have shown great promise, with the cell having the potential to cross the PCE of 20%^22^.

The first MASnI_3_ perovskite solar cell was introduced in 2014 with a PCE of 5.7%^23^. Remarkably, within the same year, Zhao improved this PCE to 6.4%^24^. Umari et al.^25^ delved into the optoelectronic properties of MASnI_3_ using a sophisticated many-body perturbation theory that factored in spin–orbit coupling. Their findings indicated that MASnI_3_ had several superior properties compared to MAPbI_3_. Specifically, MASnI_3_ showcased a charge mobility ranging from 10^2^ to 10^3^ cm^2^ V^−1^ s^−1^, a smaller direct bandgap of 1.1 eV and an increased absorption coefficient of 1.82 × 10^4^ cm^−1^ in the visible spectrum. In contrast, MAPbI_3_ had a charge mobility between 10 and 10^2^ cm^2^ V^−1^ s^−1^, a bandgap of 1.5 eV, and an absorption coefficient of 1.80 × 10^4^ cm^−1^^26^. The promising results from MASnI^3^ have propelled further interest in Sn-based perovskites. Building on this momentum, Qi et al. employed a sequential deposition technique to mitigate oxidation, achieving a notable PCE of 11.12% by strategically using a top MAX layer to prevent significant oxidation^27^. Theoretical studies of MASnI_3_-PSCs have shown that the cell has the potential to achieve a high PCE greater than 20%^28^.

Shifting the focus to germanium, Ge^2+^ shares a comparable outer ns^2^ electronic configuration with Sn^2+^ and Pb^2+^. Specifically, Ge^2+^ has a 4s^2^ structure, while Sn^2+^ and Pb^2+^ have 5s^2^ and 6s^2^ configurations, respectively. However, Ge^2+^ has a smaller ionic radius than both Sn^2+^ and Pb^2+^^29^. Ge-based perovskites such as MAGeI_3_, CsGeI_3_, and FAGeI_3_ have demonstrated stability at temperatures up to 150 °C. Given these attributes, Mathews et al. proposed that Ge could be a potential substitute for Pb in the creation of new lead-free perovskite materials. Further research by Kanatzidis et al. explored a series of AGeI_3_ perovskite compounds, assessing their structural, electronic, and optical properties^30^. They discovered that the bandgaps of Ge-based perovskites varied depending on the radius of the AGeI_3_ demonstrated high optical absorption coefficient, suggesting significant potential for photovoltaic applications. Mathews et al. fabricated the first Ge based PSC with MAGeI_3_ and CsGeI_3_ achieving current densities of 4.0 and 5.7 mA cm^−2^ and PCEs of 0.11% and 0.2%, respectively. Lastly, Kopacic et al.^31^ demonstrated that modifying the chemical composition of MAGeI_3_ could significantly boost solar cell performance and stability. By partially substituting iodide with bromide anion, they achieved a PCE of 0.57% with MAGeI_2.7_Br_0.3_. The low efficiency is attributed due to the challenging task of stabilizing Ge^2+^ in Ge-based perovskite. For this reason, very few studies have focused on fabrication on Ge based PSC. However, theoretical studies have shown that if the stability issues are overcome, the Ge-PSC can achieve PCE beyond 20%. Kanoun et al.^32^ explored MAGeI_3_ through numerical modeling and attained the PCE of 21.6%. In another study, Singh et al.^33^ also focused on Ge based cells modelling, achieving an outstanding PCE of 26.3%.

**Figure 1:**
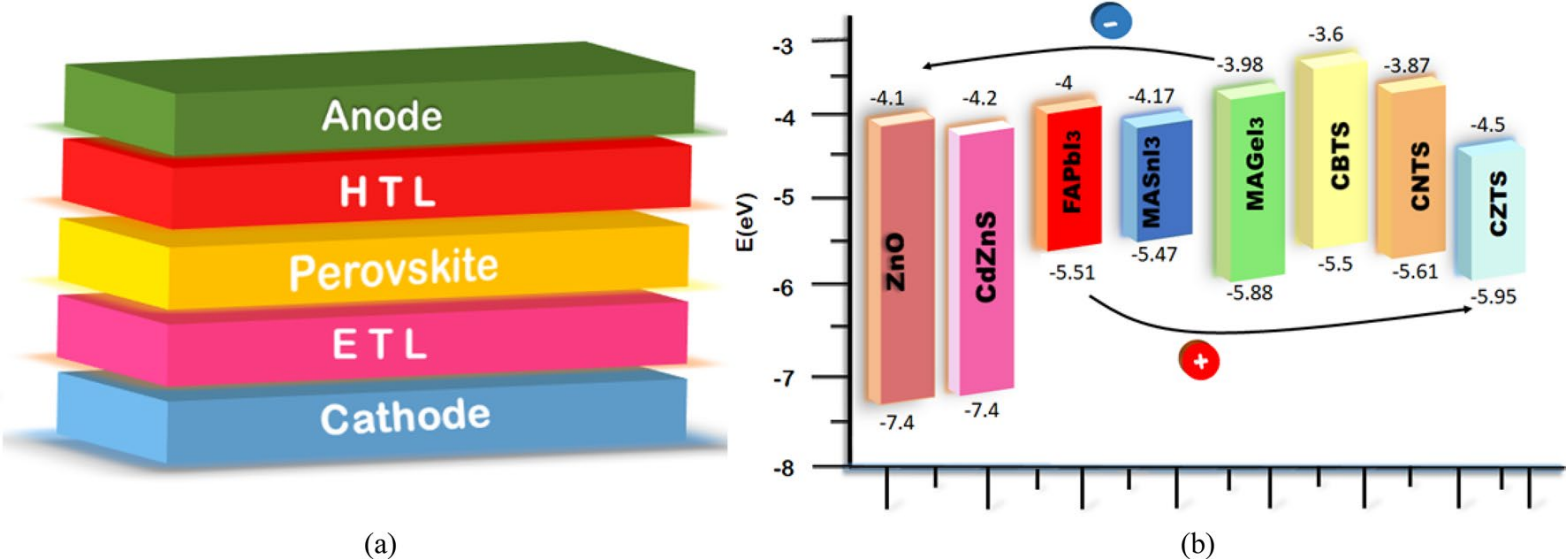
(**a**) PSC Structure. (**b**) Energy Level of Materials.

The charge transport layers (CTL) can be used to boost the performance of PSCs. The CTL are utilized to separate and collect the photo-generated charge carriers from the perovskite material^34^. A heavy donor-doped CTL is called an electron transport layer (ETL) while a heavy acceptor-doped CTL is known as a hole transport layer (HTL)^35^. The ETL and HTL are placed on either side of the perovskite material to produce two hetero-junctions^36^. Both hetero-junctions produce strong electric potential which separate the photo-generated charge carriers through their fields^37^. The ETL transports the electrons from the perovskite to the cathode, while the HTL transports the holes to the anode. Figure 1a shows the PSC structure.

The most commonly used and popular CTL for MAPbI_3_-based PSCs are the TiO_2_ (ETL) and Spiro-OMeTAD (HTL). However, both materials have their limitations and drawbacks. Spiro-OMeTD is expensive and has complex synthesis^38^. Furthermore, it tends to absorb moisture from the surroundings when exposed to the open environment, which dissolves the organic component and breaks down the precursor. Similarly, TiO_2_ produces oxygen vacancies on exposure to UV and has low charge mobility. This has led to the exploration of alternate materials for CTL^39^. Conductive materials with low resistance as CTL on the perovskite film surface have been shown to improve the PCE of the cells significantly. Raoui et al.^40^ showed that by using different ETLs (SnO_2_ & ZnO_2_) and HTLs (CuSbS_2_, Cu_2_O & CuSCN) with Pb-PSC, improved PCE between 19.7 and 26.74%. Similarly, Shasti et al.^41^ demonstrated that by replacing spriro-OMeTAD with Cu_2_O, SrCu_2_O_2_ or CuAlO_2_ as HTL produced better results with the PCE ranging between 19.67% and 19.82%.

Over the past few years, kesterite materials have emerged as a popular alternate in thin films due to their excellent PV properties^42^. Kesterites are a class of sulphide-based semiconductors that have the chemical formula of Cu_2_XSnS (CXTS). Similar to the perovskites, the kesterites have a tunable nature by changing the X in its structure with alternatives (Ba, Ni, or Zn)^43^. The kesterites are low-cost, abundant, toxic-free, highly conductive, and thermally stable. These properties make them an ideal candidate to be considered as HTL in PSC^44^. In a study by Khattak et al.^35^ six different kesterite materials were utilized as HTL with MAPbI_3_ with multiple cells achieving PCE beyond 20%. The study concluded that with proper cell design the kesterite material had high potential due to their conductive nature. In another study by Trifiletti et al. they successfully fabricated MAPbI_3_ with CZTS kesterite in both inverted and planar structure. The study concluded that the HTL performed better in planar structure due to its small band gap, achieving a PCE of 14%^45^.

Similarly, zinc-based ETLs are wide-bandgap materials with high transparency, conductivity, and stability, placing them as an alternate ETL candidate^46^. Karimi et al.^34^ compared the performance of SnO_2_ and ZnO ETL with MAPbI_3_ PSC. The study showed that after optimizing the design parameters, the ZnO based PSC outperformed its counterpart by achieving a PCE of 21.8%. Similarly, Azri et al. compared the performance of TiO_2_ and ZnO in MAPBI_3_ based PSCs. The ZnO based PSC not only showed a higher PCE (20.6%) but also demonstrated better stability with rise at interface defects^47^.

The exceptional PV characteristics, adjustable bandgap, and inherent conductivity exhibited by these materials (FAPbI_3_, MAGeI_3_, MASnI_3_, kesterites and zinc CTL) offer promising prospects for enhancing the efficiency of PSCs. A more extensive investigation into these materials and their prospective impacts holds the potential to drive innovations in PSC technology. However, the compatibility of these materials plays a significant role in the performance of the PSC. The behavior of a particular CTL may exhibit variations across the different perovskite materials^48^. This is because the different perovskite materials have varying band gaps and electron affinities. Therefore, they produce contrasting band alignment, valance band offset (VBO), conduction band offset (CBO), electric field, and recombination rate with a single CTL^41^. Raoui et al.^40^ utilized SnO_2_ and CuI as CTL with MAPbI_3_ which formed a good energy alignment and produced a PCE of 19.7%. However, when the same CTL were used with MASnI_3_ by Nine et al. the cell produced large band offsets which led to a low PCE of 9%^48^. In conclusion, the selection of CTL for a novel perovskite material cannot solely rely on its performance with a different perovskite. Understanding the CTL compatibility with the various perovskite materials holds the key to unlocking the untapped potential of the PSC. Table 1 highlights the key findings of the studies discussed in this paper.

**Table 1:** Key findings from PSC studies.

Author	PSC structure	Performance	Key findings	References
Hao et al.	MASnI_3_	PCE of 5.7%	First Successful Fabrication	^23^
Zhao et al.	MASnI_3_	Improved PCE to 6.4%	Increasing stability b reducing defects	^24^
Umari et al.	MASnI_3_	Superior properties of MASnI_3_ compared to MAPbI_3_	Many-body perturbation theory	^25^
Qi et al.	MASnI_3_	PCE of 11.12%	Sequential deposition and Oxidation mitigation	^27^
Mathews et al.	Ge-based	MAGeI_3_ and CsGeI_3_ PSCs with PCEs of 0.11% and 0.2% respectively	Proposed Ge as a potential substitute for Pb	^30^
Kopacic et al.	MAGeI_2.7_Br_0.3_	PCE of 0.57%	Chemical composition modification with Br	^31^
Kanoun et al.	MAGeI_3_	PCE of 21.6% through numerical modeling	Numerical Modelling	^32^
Singh et al.	MAGeI_3_	PCE of 26.3%	Numerical Modelling	^33^
Raoui et al.	MAPbI_3_	Improved PCE between 19.7 and 26.74% using different ETLs and HTLs	Material substitution (ETL = SnO_2_ & ZnO_2_ and HTLs = CuSbS_2_, Cu_2_O & CuSCN)	^40^
Shasti et al.	MAPbI_3_	Improved PCE between 19.67 and 19.82% using different HTLs	Replacing spriro-OMeTAD with Cu_2_O, SrCu_2_O_2_ and CuAlO_2_	^41^
Khattak et al.	MAPbI_3_	Multiple cells PCE beyond 20%	Kesterites utilized as HTL	^35^
Trifiletti et al.	MAPbI_3_	PCE of 14% using CZTS kesterite in planar structure	CZTS compared in planar and inverted structure	^45^
Karimi et al.	MAPbI_3_	Higher PCE of 21.8% with ZnO	SnO_2_ and ZnO compared as ETL	^34^
Arzi et al.	MAPBI_3_	Higher PCE 20.6% with ZnO	TiO_2_ and ZnO compared as ETL	^47^
Raoui et al. and Nine et al.	MAPbI_3_ & MASnI_3_	MAPbI_3_ achieved PCE of 19.7% while MASnI_3_ achieved 9% using SnO_2_ and CuI as CTL	MAPbI_3_ formed good band alignment while MASnI_3_ formed alignment with offsets	^48^

In this work, the correlation between the different perovskite materials and the CTLs is explored and analyzed in detail. The study focuses on the new emerging alternative materials of perovskite and CTLs. Three different perovskites of FAPbI_3_, MASnI_3,_ and MAGeI_3_ are selected along with kesterite-based HTLs (CBTS, CNTS, and CZTS) and zinc-based ETLs (ZnO and CdZnS). A total of 18 unique planar (n-i-p) PSCs are modeled in SCAPS-1D and analyzed in detail. The factors that contribute to the performance and stability of the PSC such as absorption characteristics, energy band-alignment, electric field, recombination rate, VBO/CBO, thickness, defects, and electrode work functions have been studied in detail. By conducting the investigation valuable insight can be gained into the working mechanism behind the PSC.

The evolution of PSCs has been extensively documented from their inception to their current state-of-the-art configurations. While these studies have provided invaluable insights into the individual characteristics of PSCs and CTLs, there remains a gap in our understanding of the intricate dynamics between various perovskite materials and CTLs. Many of the extant investigations have been narrowly focused, often delving deep into the singular attributes of specific perovskite materials or CTLs. This has left a void in the comprehensive study of the myriad potential combinations of these materials and their collective impact on PSC performance.

Recognizing this gap, this work aims to thoroughly examine this unexplored area. The study focuses on analyzing the PSC behavior due to the combination of different perovskite materials with various CTL. The analysis carried out seeks to unravel the underlying mechanisms that dictate the performance and outcomes of these combinations. By shining a light on the pivotal role of material compatibility, this study not only offers a fresh perspective on the current state of PSC research but also charts a clear course for future investigations. The findings of this study underscore the importance of a harmonious compatibility between perovskite materials and CTLs, emphasizing that the true potential of PSCs can only be unlocked when these components are in perfect alignment.

## Methodology

In this study, a systematic methodology is followed from our previous work^49–55^. For the absorbers, the perovskite materials of FAPbI_3_, MASnI_3,_ and MAGeI_3_ are utilized separately as the active layers in PSCs. To analyze the compatibility of CTL and its effects, three different kesterite materials of CBTS, CNTS, and CZTS are investigated as HTLs along with two different zinc-based materials of ZnO and CdZnS as ETLs. The energy levels of the materials are shown in Fig. 1b, while the design parameters are presented in the supplementary data (Table S1). The values of Table S1 are collected from an extensive literature review based on experimentation as well as density function theory calculations and numerical modeling of PSCs^43, 44, 49–51, 53, 54, 56–58^.

To ensure a strong correspondence between the study outcomes and real data, defect layers were added into the bulk of the materials. In this investigation, a defect density (Nt) of 1 × 10^14^ cm^−3^ was modeled into the bulk of the three perovskite materials along with the 5 CTL^35^. Moreover, the modeling process took into account the defects present at the interface between the materials, aiming to capture outcomes that better mirror real-world scenarios^22^. By introducing all the different defects in the bulks and at interfaces, the study sought to accommodate possible imperfections and variations from the ideal material properties, thereby yielding more realistic results.

The simulations of the cells were carried out utilizing the SCAPS-1D software. The simulation tool is renowned for its precision in modeling and analysis. All structures were simulated in compliance with standard test conditions (STC). The conditions were Air Mass of 1.5G, irradiation of 1000 W/m^2^, and Temperature of 300 K. By inputting the structural configuration and the designated conditions into SCAPS, a range of important performance parameters could be computationally ascertained. These include the current–voltage (IV) characteristics, carrier density, band diagram, current density, and several other significant performance metrics. The outcomes were derived through the solution of differential equations (Supplementary Data File—Section 2. SCAPS-1D Equations) that effectively encapsulate the behaviors of charge carriers within the perovskite solar cell architecture.

## Material compatibility

### Metal work function

The outermost layers in the PSC are the electrodes. The electrodes act as the collection point of photo-generated charge carriers in the structures^59^. For electron collection from the ETL, a cathode is used while for the hole collection from the HTL, an anode is used. The electrodes should have low resistance and high conductivity to ensure minimum energy losses due to resistive heating^60^. This is essential for maintaining a high fill FF and maximizing the PCE of the PSC. Furthermore, the compatibility of the electrodes with the CTL is of significant importance as it plays a major role in the performance of the PSC.

**Figure 2:**
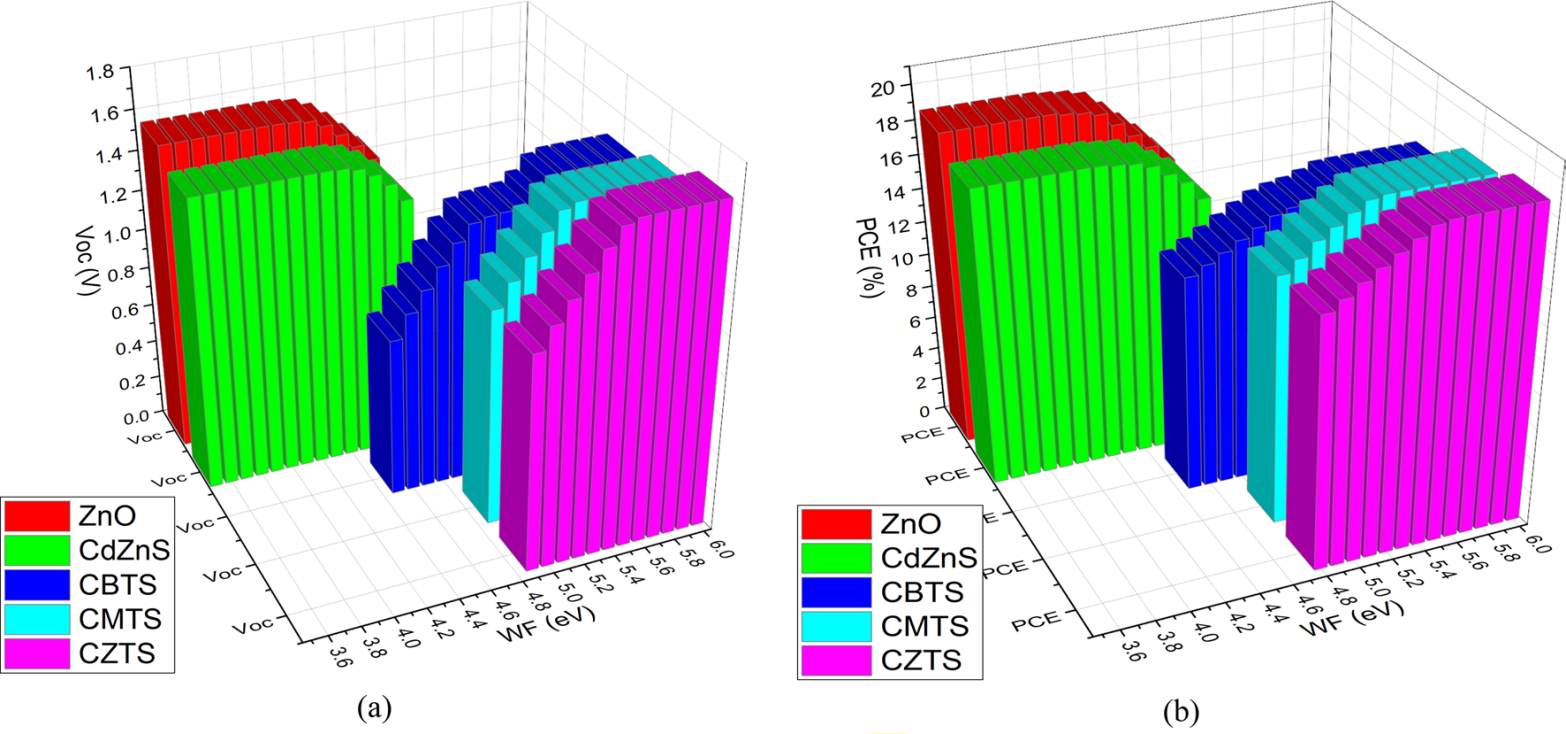
Effect of varying Work Function on the (**a**) V_oc_ (**b**) PCE of the different CTL-PSC.

The work function of an electrode characterizes its ability to either release or capture charge carriers when it is brought into contact with another material^61^. It is the energy required to move a charge carrier from the highest occupied energy level (Fermi level) of the material to a reference energy level. The work function difference between the electrode and the adjacent CTLs influences the ease with which the charge carrier can be extracted from the perovskite layer^62^. For efficient charge extraction, the electrode work function should align with the energy level of the CTL. Adequate alignment minimizes energy barriers, thus reducing the likelihood of charge recombination^63^. An appropriate work function helps maximize the open circuit voltage (V_oc_) and power conversion efficiency (PCE) of the PV cell. Varying work function values were checked with the different CTLs to identify the most compatible one. Figure 2 shows the effect of different work functions on the performance of the CTL.

The results in Fig. 2 show contrasting behavior between the ETL and HTL. The ETL performance improves by reducing the value of the work function. It saturates near 4.4 eV and further reduction does not affect the performance. On the other hand, the performance of the HTL improves with an increase in the work function. The saturation is achieved near 5.2 eV. The difference in behavior of the two CTLs is because of the presence of charge carriers in different energy bands. In the ETL, the electrons are present in the conduction band which occupies a lower energy level^64^. In the HTL, the holes are present in the valance band which occupies a higher energy level^65^. For the ETL, materials with low work function, preferably below 4.4 eV, are compatible. While for the HTL, materials of work function above 5 eV are suitable. The different electrode materials have varying work functions. Table S2 presents different conductive materials along with their work function. The transparent conductive oxide (TCO) glass can also be used as electrodes. The TCO’s work function can be tuned over a large range by treating it with chloride and fluoride^66^.

### Absorption & transmissivity

Materials characterized by wide band gaps (E_g_) typically exhibit high optical transmissivity and low absorption^49^. Conversely, materials featuring narrow band gaps tend to possess low transmissivity and high absorption. This correlation between band gap and absorption (A) is defined by Eq. (1), while the connection between absorption and transmissivity (T) is expressed by Eq. (2).

1 $$\left( {\alpha *{\text{hv}}} \right)^{{{1}/\gamma }} = {\text{B}}\left( {{\text{hv}} - {\text{E}}_{{\text{g}}} } \right)$$

2 $$A= {-\mathrm{log}}_{10}T$$

where α = absorption-coefficient, h = plank-constant, v = photon-frequency, γ = direct/indirect transition, and B = energy independent constant.

About 90% of the electromagnetic light spectrum is encompassed within the visible and infrared ranges^67^. The UV spectrum causes the degradation of encapsulants and materials while the high infrared spectrum builds up heat in the material. Both these spectrums are usually not useful in PV. The visible light spectrum which spans from approximately 380 nm (violet light) to 750 nm (red light) is the most useful and targeted spectrum^68^. This has been the base for the strategic design of numerous commercial photovoltaic modules which have been purposefully tailored to harness and effectively utilize the inherent energy present within the visible spectrum of light.

**Figure 3:**
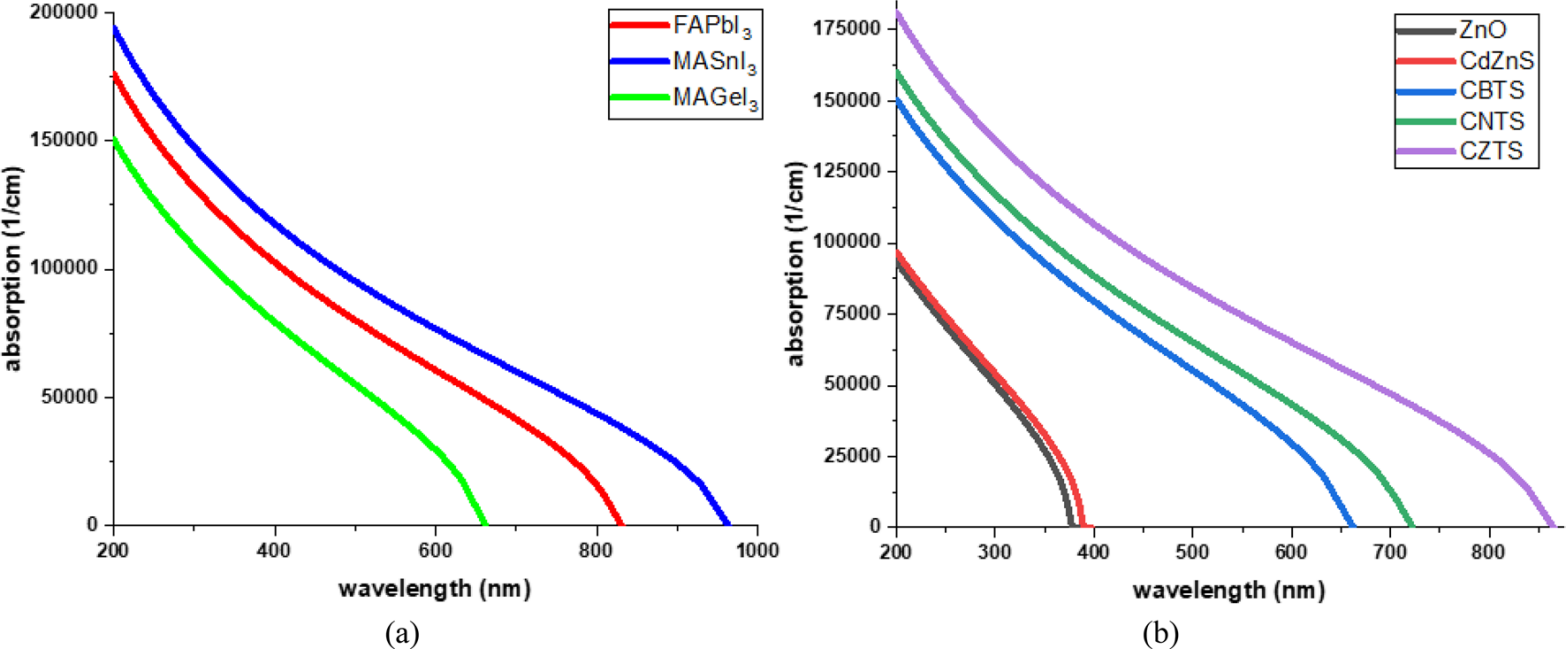
Optical Absorption of the (**a**) Perovskite Materials (**b**) CTL.

Figure 3a represents the optical absorption of the selected perovskite materials which have different band gaps. The results show that all three perovskite materials have high absorption in the visible spectrum. MASnI_3_ has the smallest band gap coinciding with the largest optical absorption and least transmittivity. While MAGeI_3_ has the largest band gap with the lowest absorption and largest transmittivity. However, the absorption range of MAGeI_3_ still covers the majority of the visible spectrum, having a cut-off beyond 660 nm. This property can be exploited by the semi-transparent PSCs where partial optical transmissivity is a requirement.

In the p-i-n structure, the ETL serves as the front layer through which light penetrates to reach the perovskite layer, while the HTL is positioned as the rare layer. The ETL being the front layer should exhibit high photon transmissivity coupled with low photon absorption. This arrangement would ensure that maximum photons reach the perovskite material, enabling efficient energy conversion. Not all the photons are absorbed by the absorber layer. Some photons, including those of low band energy and large wavelength, pass the absorber without being absorbed. Interestingly, when HTL of band gap smaller than the absorber material are used, they can inadvertently contribute to the optical absorption of these lower energy photons^50^.

Figure 3b shows the optical absorption of the CTL. Both the ZnO and CdZnS have low optical absorption in the visible spectrum due to their large band gap. This makes them ideal to be used as the front layers (ETLs) in the PSCs as they have high optical transmissivity. The kesterite-based materials have high optical absorption due to their small band gaps. The CZTS has a band gap smaller than FAPbI_3_ and MAGeI_3_, therefore it has absorption higher than them. Utilizing CZTS with these perovskites may contribute to boosting the PSCs’ photo generation.

### Energy band alignment

The alignment of energy bands across the various layers within a PSC structure plays an important role in dictating its overall performance. The degree of conduction band alignment between two materials determines the electron flow^41^. Linear alignment ensures the charge carriers' flow while a blockage is produced due to offset. Similarly, the alignment of valence bands between two materials determines hole flow. To achieve high operational efficiency, ideal energy band alignment requires minimal Conduction Band Offset (CBO) and maximal Valence Band Offset (VBO) between the perovskite and ETL^44^. Such alignment ensures the movement of a maximum number of electrons from the perovskite to the ETL, while also blocking the mobility of holes. Similarly, the alignment between the perovskite and HTL should emphasize minimal VBO and maximal CBO, ensuring the efficient movement of holes from the perovskite to the HTL, while concurrently blocking electron movement.

The band alignment of these structures is determined by the individual E_g_ and electron affinity (χ) of each material, as described by Eqs. (3) and (4):

3 $${\text{C}}.{\text{B}}.{\text{O}} = \left( {\chi_{{{\text{Abs}}}} {-}\chi_{{{\text{CTL}}}} } \right)$$

4 $${\text{V}}.{\text{B}}.{\text{O}} = \left( {\chi_{{{\text{CTL}}}} {-}\chi_{{{\text{Abs}}}} + {\text{E}}_{{{\text{g}} - {\text{CTL}}}} {-}{\text{E}}_{{{\text{g}} - {\text{Abs}}}} } \right)$$

When the perovskite layer’s conduction band (CB) is situated above the ETL’s CB at hetero-junction, it creates a cliff (-CBO)^50^. This negative CBO has an adverse impact on PSC performance because it reduces both the activation energy (E_a_) against recombination at the heterojunction and the built-in potential (V_bi_), ultimately resulting in a lower V_oc_. Conversely, when the perovskite CB is positioned below the CB of the ETL, a spike (+CBO) form^50^. This positive CBO leads to an increased V_bi_ at the heterojunction, thereby enhancing the V_oc_. However, excessively large spikes can create barriers that impede electron transport, increasing recombination due to the reduced E_a_. The optimal scenario occurs when a zero CBO is achieved, indicating a perfect alignment of conduction bands between the layers.

Similarly, when the perovskite layer’s valence band (VB) is positioned below the HTL’s VB, a cliff-like discontinuity (-VBO) forms reducing the V_bi_, thereby influencing overall efficiency^49^. Conversely, if the absorber's VB is situated above the VB of the HTL, a spike (+VBO) arises, increasing V_bi_. Similarly, to the CBO when these spikes become excessively large, they create obstacles to hole movement across the interface.

**Figure 4:**
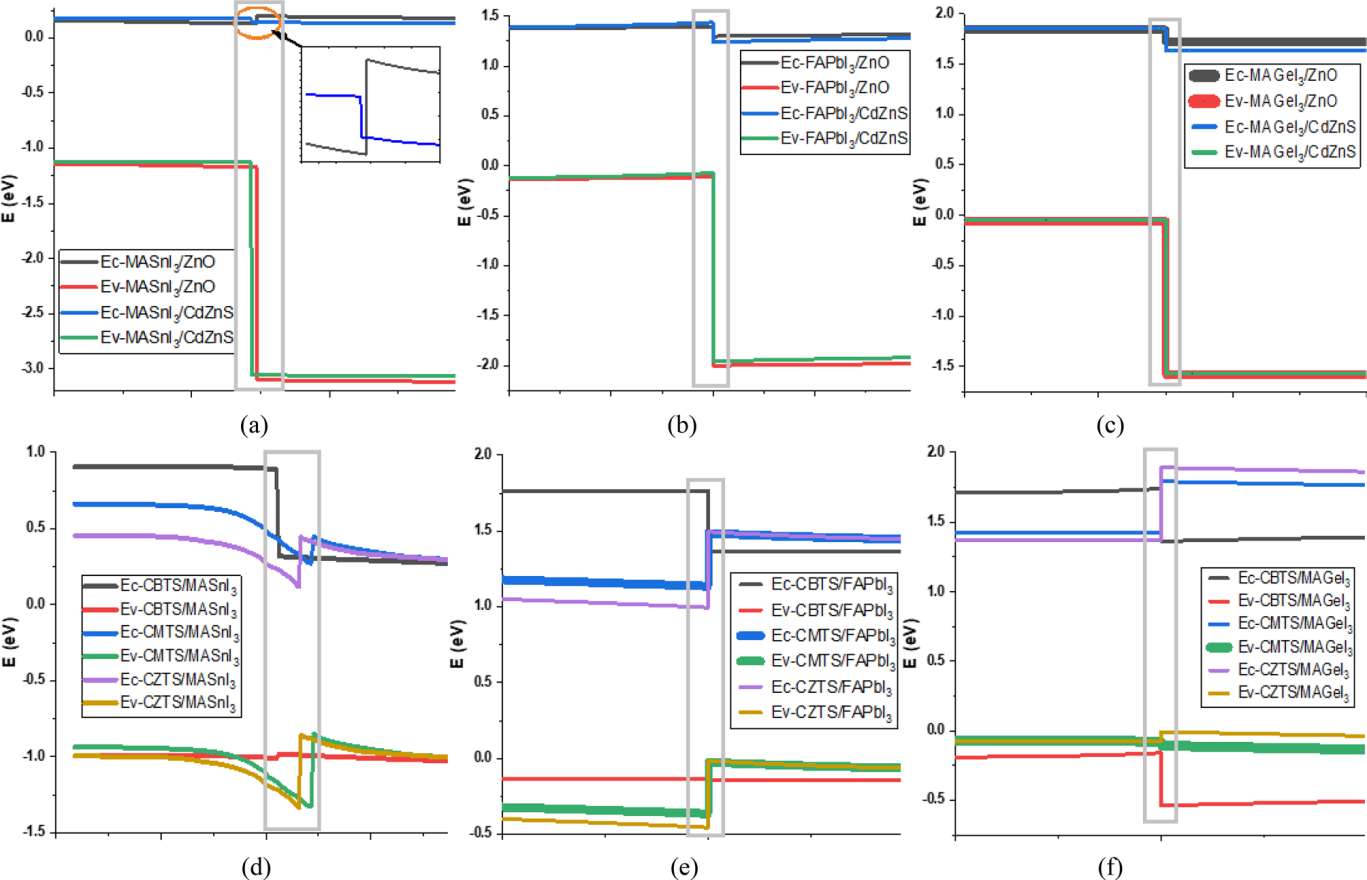
Energy Band Alignment between the (**a**) MASnI_3_ & ETL (**b**) FAPbI_3_ & ETL (**c**) MAGeI_3_ & ETL (**d**) HTL & MASnI_3_ (**e**) HTL & FAPbI_3_ (**f**) HTL & MAGeI_3_.

Figure 4 shows the energy band alignment established by the different perovskite materials with the CTLs. Notably, the same CTL can exhibit varying degrees of band offset with different perovskites, as detailed in Fig. S1 of the supplementary data. From the simulated results of the Perovskite/ETL hetero-junctions, ZnO's conduction band alignment creates a spike when paired with MASnI_3_ but forms a cliff when combined with the other two perovskites. Conversely, CdZnS generates cliffs of differing magnitudes when utilized with all three perovskites. In both cases, the ETLs result in significant VBO across all three perovskite materials.

For the HTLs, the valence band of CBTS forms a spike when utilized with MASnI_3_, a smaller cliff with FAPbI_3_, and a substantial cliff with MAGeI_3_. Notably, CBTS consistently establishes a large CBO with all three perovskites. In contrast, CMTS forms an imperfect band alignment, characterized by a small CBO and significant VBO when combined with MASnI_3_. However, it achieves better alignment with the other two perovskites. Finally, CZTS exhibits satisfactory band alignment with a small VBO spike and a substantial CBO when utilized with MAGeI_3_, yet it forms inadequate alignments characterized by large VBO spikes with the other two perovskites.

In summary, all three perovskites demonstrate acceptable band alignment with both ETLs. Among the HTLs, FAPbI_3_ exhibits the most favorable alignment with CMTS, while MASnI_3_ aligns best with CBTS, and MAGeI_3_ forms the most suitable alignment with CZTS. These band alignments play a pivotal role in the performance of PSCs, impacting charge carrier transport and recombination dynamics.

### Electric potential at hetero-junction

**Figure 5:**
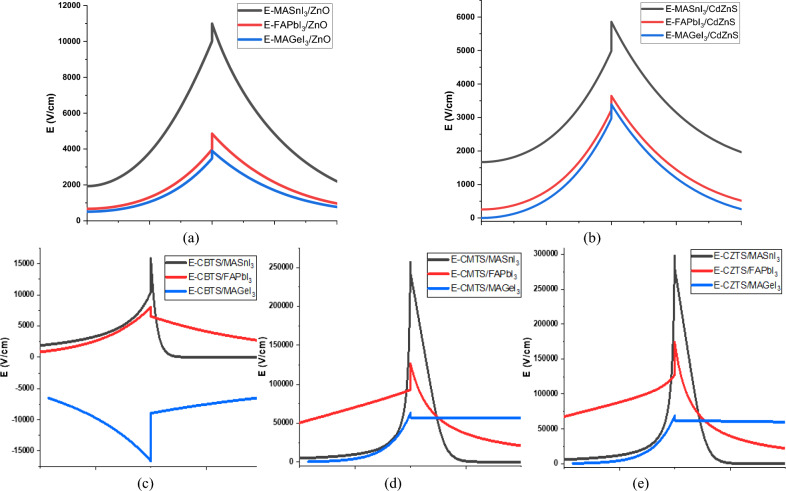
Electric Field produced between the (**a**) Perovskites & ZnO (**b**) Perovskite & CdZnS (**c**) CBTS & Perovskites (**d**) CBTS & Perovskites (**e**) CZTS & Perovskites.

The electric field at the hetero-junction is produced due to the differences in charge carrier concentrations and energy band alignments between the two materials (Perovskite/CTL)^69^. It significantly influences the V_bi_ and V_oc_ of the cell. The primary function of the electric field is to assist in driving the photo-generated charge carriers to their respective electrodes, i.e., electrons to ETL and holes to HTL^41^. It also plays a role in blocking minority charge carriers by creating a barrier for their movement toward the CTL. Figure 5 shows the electric potential formed by the different perovskite materials with the CTLs at the hetero-junction.

Conversely, in the case of the HTL/Perovskite hetero-junctions, MASnI_3_ consistently produces the highest electric field with all HTLs. This heightened electric field can be attributed to the significant spikes in VBO formed by MASnI_3_ with all the HTLs. Following it, is the electric field generated by FAPbI_3_ when paired with CZTS and CMTS, respectively, due to the smaller spikes formed by the pairing. However, MAGeI_3_ generates the weakest electric field when combined with the HTLs. This is primarily because it forms only a small spike and cliff with CZTS and CMTS respectively. Interestingly, a unique scenario unfolds with the pairing of MAGeI_3_ and CBTS. These materials create a large cliff VBO at the hetero-junction, effectively obstructing the flow of holes. Consequently, the concentration of holes increases in this region, generating its electric field in the opposite direction.

### Recombination rate at hetero-junction

**Figure 6:**
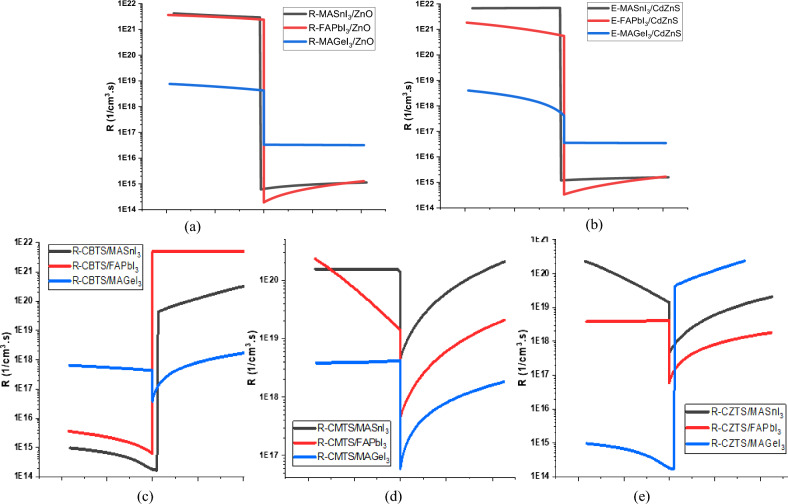
Recombination Rate at the hetero-junction of (**a**) Perovskites & ZnO (**b**) Perovskite & CdZnS (**c**) CBTS & Perovskites (d) CBTS & Perovskites (e) CZTS & Perovskites.

Recombination rate at hetero-junction refers to the rate at which photo-generated charge carriers recombine with each other at the interface between two different materials. The main factor for recombination at the hetero-junction is the inadequate band alignment between the two layers^70^. The occurrence of either the crossing over of opposite charge carriers or the obstruction of their respective carriers at the CTL results in an increased recombination rate. Conversely, a seamless transition of charge carriers and the impeding of opposite carriers reduces the recombination rate. Figure 6 shows the recombination rate at the hetero-junctions of different perovskite materials with the CTLs.

At the Perovskite/ETL hetero-junctions, a reduction in recombination rates occurs for both ETLs with all the perovskites due to the ideal band alignment. Notably, MASnI_3_ produces the most favorable alignment, resulting in the most significant reduction in recombination. In contrast, MAGeI_3_, which forms the least ideal alignment, exhibits the lowest reduction in recombination.

While at the HTL/Perovskite hetero-junctions, the utilization of CBTS with MAGeI_3_ leads to increased recombination at the hetero-junction due to their inadequate band alignment. However, with the other perovskites, CBTS aligns well, resulting in a reduction in recombination. Similarly, CZTS demonstrates a distinctive behavior. It forms an acceptable band alignment with MAGeI_3_, leading to reduced recombination, but exhibits inadequate alignment with the other two perovskites, consequently increasing recombination rates. Furthermore, due to the small CBO of CMTS with all the perovskite materials, there is a noticeable increase in recombination at the hetero-junction.

## IV Analysis

**Figure 7:**
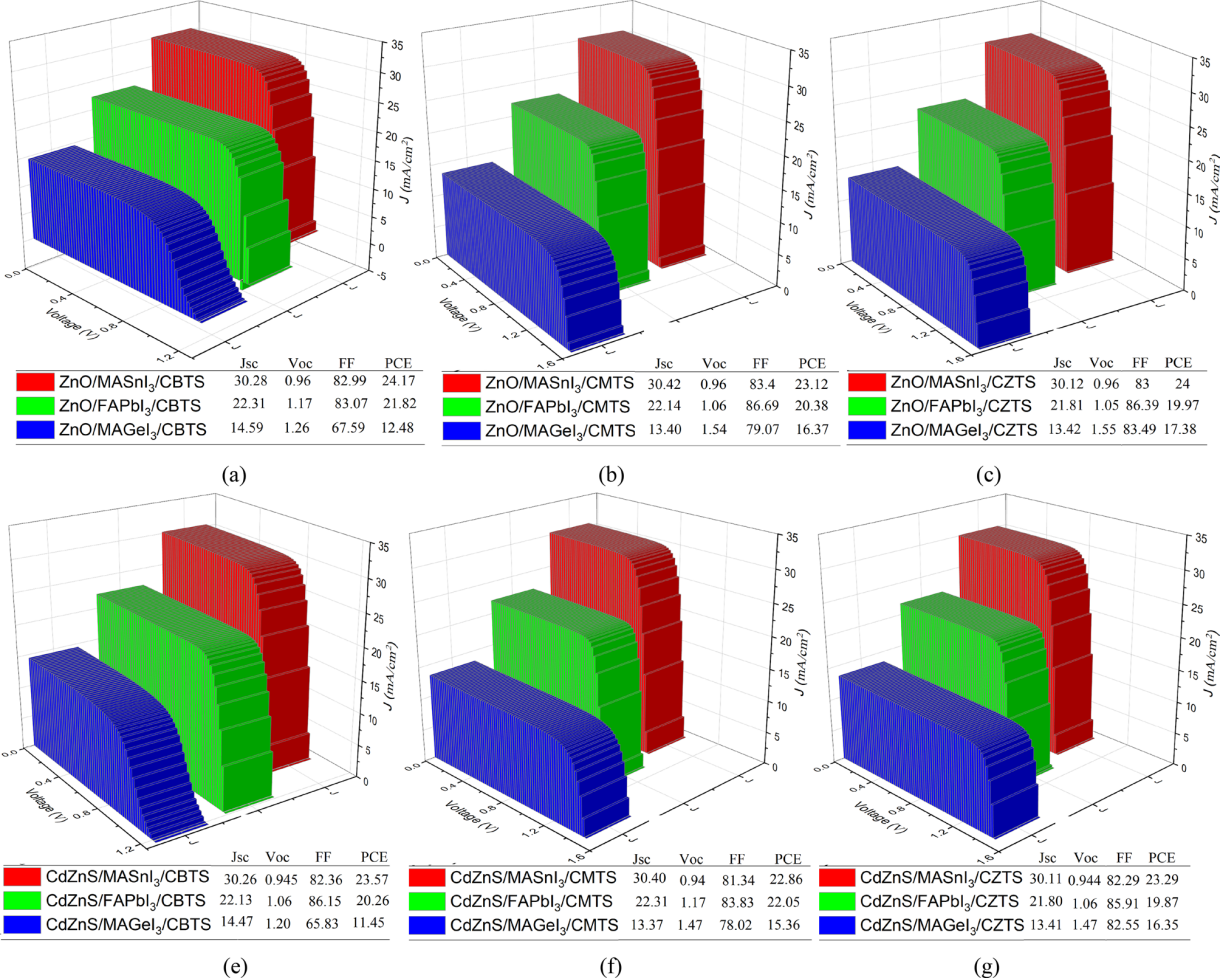
IV Characteristics of the perovskites with (a)ZnO/CBTS (b) ZnO/CMTS (c) ZnO/CZTS (**d**) CdZnS/CBTS (**e**) CdZnS/CMTS (**f**) CdZnS/CZTS.

A total of 18 unique structures were modeled from the combination of the different layers. Figure 7 shows the IV characteristics of all the structures. The MASnI_3_-based structures produce the highest short circuit current (J_sc_) due to the absorber having the lowest band gap, leading to the highest absorption coefficient. While the MAGeI_3_-based structures produced the highest V_oc_ due to the material's large band gap. The three absorbers yielded their best results when paired with distinct combinations of CTLs. For the Pb-based PSC, CdZnS/FAPbI_3_/CMTS produced the highest PCE of 22.05. Similarly, for the Ge-based PSC, ZnO/MAGeI_3_/CZTS produced the highest performance with a PCE of 17.28%. While for the Sn-based PSC, ZnO/MASnI_3_/CBTS produced the highest PCE of 24.17%.

### Perovskite thickness optimization

**Figure 8:**
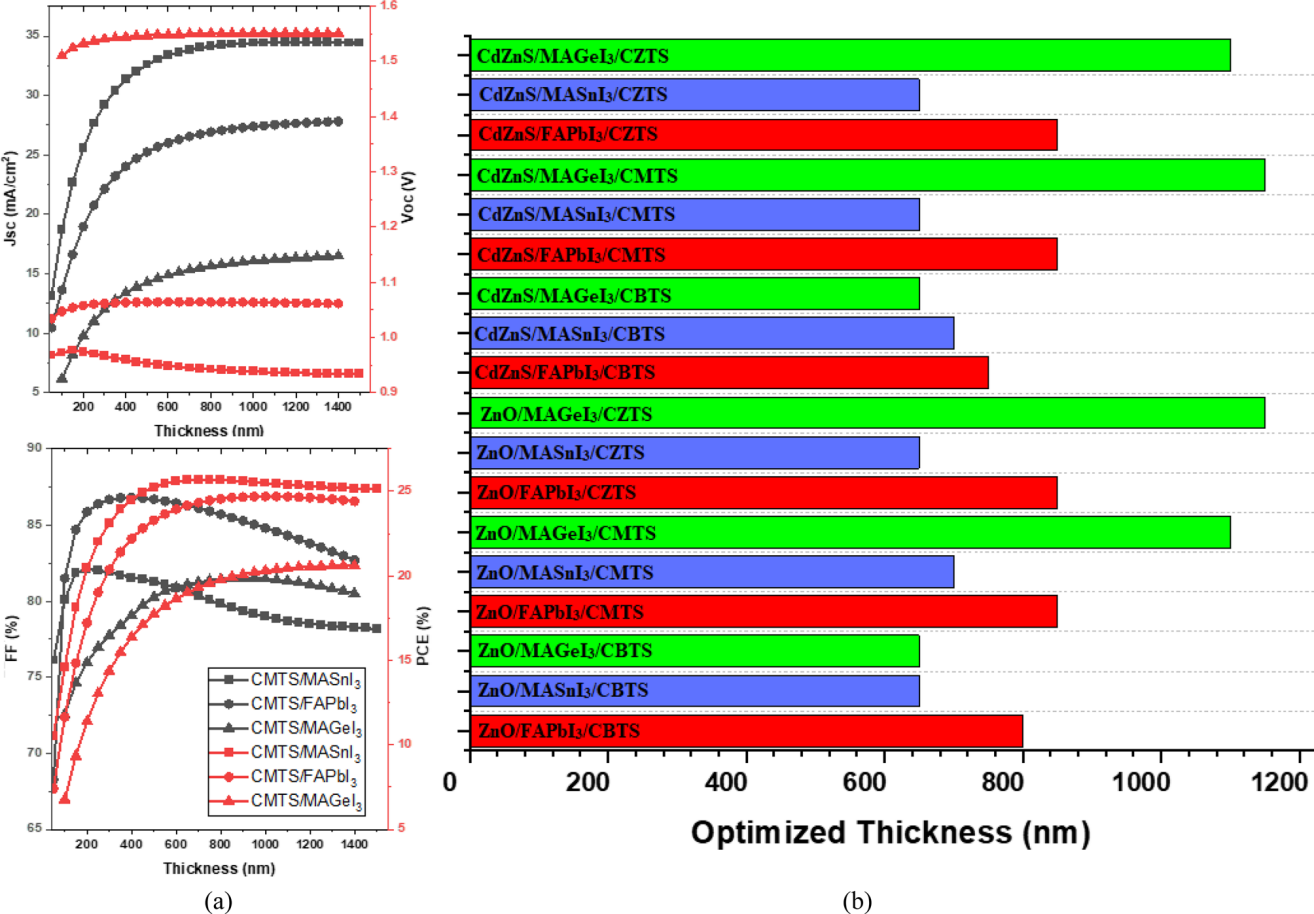
(**a**) Effect of Perovskite Thickness on performance (**b**) Optimized Thickness of the PSCs.

The thickness of the perovskite material has a significant impact on the PV properties of the PSC, such as photo generation, carrier transport, and charge collection within the cells. Figure 8a presents the effect of increasing thickness on the performance of CMTS-based PSC structures, while the results of the remaining structures are shown in Figs. S2 and S3 of the supplementary data. The varying thickness of all three perovskite materials has a profound impact on the IV characteristics of the PSC. Increasing the layer's thickness up to its optimized value leads to notable enhancements in the cell's PCE and J_sc_. This improvement occurs due to the increased photon capacity of the thicker perovskite layer, absorbing a wider range of the solar spectrum^51^. Consequently, this photogeneration boost contributes to an overall enhancement in PSC performance. However, exceeding this optimized thickness results in a decline in PCE. This decline is attributed to the fact that the layer's thickness surpasses the carrier lifetime, increasing the probability of carrier recombination^55^. The fill factor (FF) of the PSC also declines with an increase in the thickness due to the rise in the series resistance of the cell.

Figure 8b shows the optimized thickness of all the PSC structures modeled in this study. The Sn-based structures reach their optimization point relatively quickly. This can be attributed to their large absorption coefficient, due to their smaller band gap^53^. As a result, the Sn-based structures achieve their optimized thickness within the range of 650–700 nm. Conversely, the Ge-based structures require a greater thickness to reach optimization, because of their lower absorption coefficient, a consequence of their larger band gap. To attain optimization, the Ge-based structures need thicknesses surpassing 1000 nm. Meanwhile, the Pb-based structures exhibit an optimized thickness within the range of 800–850 nm.

Notably, the optimized thickness of the perovskite layer is also influenced by the choice of CTLs utilized. CTL which forms adequate band alignment with a perovskite facilitates the smooth transport of charge carriers, allowing the PSC to achieve higher optimized thickness with increased performance^49^. While inadequate band alignment hinders the transport of charge carriers, leading to lower optimized thickness and performance. For this reason, the CBTS structure with MAGeI_3_ achieves a lower optimized thickness than the other Ge-based PSCs.

### Effect of defect density

**Figure 9:**
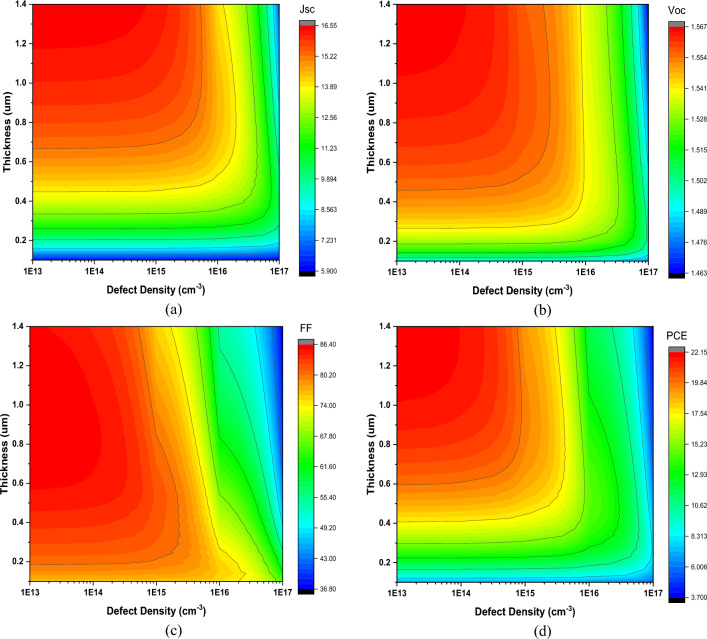
Effect of Defect Density on PSC’s Performance.

In poly-crystalline perovskite materials, the Defect Density (N_t_) typically falls within a range of 1 × 10^13^–1 × 10^17^ cm^−3^^61^. To investigate the influence of N_t_ on the performance and optimized thickness of the perovskite layer, the defect density of the perovskite material varied from 10^13^ to 10^17^ cm^−3^, and the results are shown in Fig. 9.

The N_t_ plays a significant role in determining the cell's performance. As the N_t_ increases, all the IV characteristics of the cell exhibit a reduction in performance. This is because a higher N_t_ signifies a greater number of trap layers within the perovskite material^71^. These elevated trap states impede the flow of charge carriers within the layers and cause recombination^72^. Both of these factors contribute to a decrease in the cell's overall performance. Furthermore, a higher N_t_ results in a reduction in the carrier lifetime of the photo-generated carriers. This, in turn, leads to an increase in the recombination rate and a decrease in the absorption length of the cell^73^. The cells achieve optimized thickness at a lower value with decreased output performance.

## Conclusion

In this study, three alternative perovskite materials of FAPbI_3_, MASnI_3,_ and MAGeI_3_ were focused upon along with the recently emerged kesterite (CBTS, CMTS, and CZTS) and zinc (ZnO and CdZnS) materials as CTLs. A total of 18 structures were modeled from the combination of the different layers. The performance and optimization of the different PSCs were investigated by analyzing various design parameters and their impact on the cell's output characteristics. The compatibility of the CTL with the different perovskite materials significantly affected the PSC performance and produced varying PCE. A single CTL did not produce the same results as an alternative perovskite material because of the difference in band gap and electron affinity. Both parameters affected the band alignment, band offsets, electric field, and recombination rate, leading to varying results. Large band offsets reduced the electric field at the heterojunction along with increasing the interface recombination. The degree of band alignment and band gap also significantly influenced the optimized thickness. The perovskite with a small band gap (MASnI_3_) achieved optimized thickness sooner (between 60 and 700 nm) due to a higher absorption coefficient than the perovskite with a large band gap (MAGeI_3_) (greater than 100 nm) while, FAPbI_3_ achieved optimized thickness in the range of 800 nm. Furthermore, due to inadequate band alignment, the CBTS/MAGeI_3_-based structures achieved optimized thickness at a lower value (650 nm) with decreased output performance (PCE 11–12%) than the other Ge-based structures. Interestingly, the defect density not only influenced the IV characteristics but also the optimized thickness. The increase in defect density decreased the carrier lifetime and increased trap levels in the material’s bulk which led to reduced optimized thickness along with lower performance. Furthermore, the optimal work function for each CTL were also identified. The ETL performed best with metals of work function below 4.4 eV while the HTL preferred metals with work function greater than 5.5 eV. Based on the results the most suitable CTL were CdZnS/CMTS for FAPbI_3_ producing a PCE of 22.05%, ZnO/CZTS for MAGeI_3_ with PCE of 17.28% and ZnO/CBTS for MASnI_3_ with a PCE of 24.17%. This study provides a pivotal insight into the intricate interplay between various perovskite materials and CTLs, providing a path for future endeavors aimed at maximizing the potential of these combinations.

### Supplementary Information

Supplementary Information.

## Data Availability

Data available upon reasonable request from the corresponding author Dr. Muhammad Noman (muhammad.noman@uetpeshawar.edu.pk).
