# Supplementary material for: Comprehensive analysis of heterojunction compatibility of various perovskite solar cells with promising charge transport materials
Source: Sci Rep. 2023 Nov 3;13:19015. doi: 10.1038/s41598-023-46482-5 (PMC10624924; doi:10.1038/s41598-023-46482-5)
Supplement: Supplementary file 1 — Supplementary Information. [file 41598_2023_46482_MOESM1_ESM.docx]

**Supplementary Data**

1. **Design Parameters of the Layers**

Table S1 shows the design parameters of the three perovskite materials, two zinc ETLs and three HTLs used in this study to model the different PSC structures.

Table S1. Design Parameters of Materials

|  | **ZnO** | **CdZnS** | **FAPbI_3_** | **MASnI_3_** |
| --- | --- | --- | --- | --- |
| Thickness (nm) | 150 | 150 | 400 | 400 |
| Bandgap (eV) | 3.3 | 3.2 | 1.51 | 1.3 |
| Electron Affinity (eV) | 4.1 | 4.2 | 4 | 4.17 |
| Dielectric Permittivity | 9 | 9.12 | 6.6 | 8.2 |
| CB Effective Density of State(cm^-3^) | 4X10^18^ | 1.5X10^18^ | 1.2X10^19^ | 1X10^18^ |
| VB Effective Density of State(cm^-3^) | 1X10^19^ | 1.8X10^19^ | 2.9X10^18^ | 1X10^18^ |
| Electron Mobility (cm^2^/Vs) | 100 | 250 | 2.7 | 1.6 |
| Hole Mobility (cm^2^/Vs) | 25 | 40 | 1.8 | 1.6 |
| Donor Density N_D_ (cm^-3^) | 1X10^16^ | 1X10^16^ | 1X10^9^ | 1X10^14^ |
| Acceptor Density N_A_ (cm^-3^) | - | - | 1X10^9^ | 1X10^14^ |
|  | **MAGeI_3_** | **CBTS** | **CNTS** | **CZTS** |
| Thickness (nm) | 400 | 150 | 150 | 150 |
| Bandgap (eV) | 1.9 | 1.9 | 1.74 | 1.45 |
| Electron Affinity (eV) | 3.98 | 3.6 | 3.87 | 4.5 |
| Dielectric Permittivity | 10 | 5.4 | 9 | 9 |
| CB Effective Density of State(cm^-3^) | 1x10^16^ | 2.2X10^18^ | 2.2X10^18^ | 2.2X10^18^ |
| VB Effective Density of State(cm^-3^) | 1x10^15^ | 1.8X10^19^ | 1.8X10^19^ | 1.8X10^19^ |
| Electron Mobility (cm^2^/Vs) | 16.2 | 30 | 11 | 60 |
| Hole Mobility (cm^2^/Vs) | 10.1 | 10 | 11 | 20 |
| Acceptor Density N_A_ (cm^-3^) | 1x10^9^ | - | - | - |
| Acceptor Density N_A_ (cm^-3^) | 1x10^9^ | 1X10^15^ | 1X10^16^ | 1X10^18^ |

Table S2 shows the work function values of the different materials that can be utilized as electrodes in the PSCs

.

Table S2.

| **Materials** | **Al** | **Cu** | **Ag** | **Fe** | **C** | **Au** | **Pd** | **Ni** | **Pt** |
| --- | --- | --- | --- | --- | --- | --- | --- | --- | --- |
| $\boldsymbol{\emptyset}$ **(eV)** | 4.3 | 4.65 | 4.7 | 4.8 | 5 | 5.1 | 5.3 | 5.5 | 5.65 |

1. **SCAPS-1D Equations**

The poison equation governs the electric field of a p-n junction $(E)$ and the charge density $(\rho)$ in the semiconductor material and is given by:

|  | $\frac{\partial^{2}\varphi}{\partial^{2}x}=-\frac{\partial E}{\partial x}=-\frac{\rho}{\varepsilon_{S}}=-\frac{q}{\varepsilon_{S}} [p-n N_{D}^{+}\left( x \right)- N_{A}^{-}\pm N_{def}(x)$ | (1) |
| --- | --- | --- |

Where $\varphi$ = electrostatic potential, *q =* elementary charge, $\varepsilon_{S}$*=* relative permittivity, n & p = electron and hole density respectively, are $N_{D}^{+}$ = donor density, $N_{A}^{-}$ *=* acceptors density, and $N_{def}$ = defect density due to trapped charged carriers.

The continuity equation governs the generation, recombination, drift and diffusion mechanism of charge carrier concentration and is given by:

|  | $\frac{ⅆp_{n}}{ⅆt}=G_{p}-\frac{p_{n}-p_{n0}}{\tau_{p}} + p_{n}\mu_{p}\frac{d\xi}{dx}+ \mu_{p}\xi\frac{ⅆp_{n}}{ⅆx}+ D_{p}\frac{ⅆ^{2}p_{n}}{ⅆx^{2}}$ | (2) |
| --- | --- | --- |
|  |  |  |
|  | $\frac{ⅆn_{p}}{ⅆt}=G_{n}-\frac{n_{p}-n_{p0}}{\tau_{n}} + n_{p}\mu_{n}\frac{d\xi}{dx}+ \mu_{n}\xi\frac{ⅆn_{p}}{ⅆx}+ D_{n}\frac{ⅆ^{2}n_{p}}{ⅆx^{2}}$ | (3) |

Where$G_{n}$, $G_{p}$ = generation rate of charge carriers, $\xi$ = electric field, $\mu_{n}$ & $\mu_{p}$ = carrier mobilities, $D_{n}$ & $D_{p}$ = carrier diffusion coefficient, $\tau_{n}$& $\tau_{p}$ = carrier lifetime.

Carrier transport occurs by drift and diffusion and is given by:

|  | $J_{n}=qn\mu_{n}E+qD_{n} \frac{\partial n}{\partial x}$ | (4) |
| --- | --- | --- |
|  |  |  |
|  | $J_{p}=qn\mu_{p}E+qD_{p} \frac{\partial p}{\partial x}$ | (5) |

Where $J_{n}$ and $J_{p}$ = carriers’ current densities.

Open circuit Voltage:

|  | $V_{OC}= \frac{nkT}{q}[ln(\frac{I_{L}}{I_{O}}+1)]$ | (6) |
| --- | --- | --- |

Where $\frac{kT}{q}$ = thermal voltage, $I_{L}$ = light-generated current and $I_{O}$ = saturation current.

Fill Factor:

|  | $FF=\frac{P_{max}}{P_{t}}=\frac{V_{max} I_{max}}{V_{OC} I_{SC}}$ | (7) |
| --- | --- | --- |

Power Conversion Efficiency:

|  | $PCE=\frac{V_{OC} I_{SC} FF}{P_{in}}$ | (8) |
| --- | --- | --- |

1. **Band Offset Values**

Figure S1 shows the conduction band offset and valance band offset formed by the different charge transport layers with the three distinct perovskite materials.

Figure S1. VBO and CBO of CTL with the Perovskites.

1. **Optimized Thickness Values**

Figure S2 shows the effect of the zinc ETLs and absorber thickness on the performance of the PSC. While S2 show the effect of kesterite HTLs on the performance.

|  |  |
| --- | --- |
|  |  |

Figure S2. Effect of zinc ETLs and absorber thickness on the performance of the PSC

|  |  |
| --- | --- |
|  |  |
|  |  |

Figure S3. Effect of Kesterite HTLs and absorber thickness on the performance of the PSC
